# Evaluation of the Diabetes Screening Component of a National Cardiovascular Risk Assessment Programme in England: a Retrospective Cohort Study

**DOI:** 10.1038/s41598-020-58033-3

**Published:** 2020-01-27

**Authors:** Raffaele Palladino, Eszter P. Vamos, Kiara Chu-Mei Chang, Kamlesh Khunti, Azeem Majeed, Christopher Millett

**Affiliations:** 1 Public Health Policy Evaluation Unit, School of Public Health, Imperial College London, London, United Kingdom; 2 Department of Public Health, University, “Federico II” of Naples, Naples, Italy; 3 Diabetes Research Centre, Leicester Diabetes Centre, University of Leicester, Leicester, United Kingdom; 4 Department of Primary Care and Public Health, Imperial College London, London, United Kingdom

**Keywords:** Endocrine system and metabolic diseases, Health services, Epidemiology

## Abstract

Type 2 Diabetes (T2D) is increasing but the effectiveness of large-scale diabetes screening programmes is debated. We assessed associations between coverage of a national cardiovascular and diabetes risk assessment programme in England (NHS Health Check) and detection and management of incident cases of non-diabetic hyperglycaemia (NDH) and T2D. Retrospective analysis employing propensity score covariate adjustment method of prospectively collected data of 348,987 individuals aged 40–74 years and registered with 455 general practices in England (January 2009-May 2016). We examined differences in diagnosis of NDH and T2D, and changes in blood glucose levels and cardiovascular risk score between individuals registered with general practices with different levels (tertiles) of programme coverage. Over the study period 7,126 cases of NDH and 12,171 cases of T2D were detected. Compared with low coverage practices, incidence rate of detection in medium and high coverage practices were 15% and 19% higher for NDH and 10% and 9% higher for T2D, respectively. Individuals with NDH in high coverage practices had 0.2 mmol/L lower mean fasting plasma glucose and 0.9% lower cardiovascular risk score at follow-up. General practices actively participating in the programme had higher detection of NDH and T2D and improved management of blood glucose and cardiovascular risk factors.

## Introduction

Diabetes is rising globally, with projections suggesting that the number of adults with the condition will increase from 415 million to 642 million between 2015 and 2040^1,2^. Many cases, estimated to be 175 million worldwide, are currently undiagnosed^3^ and 230 million people are estimated to have non-diabetic hyperglycaemia (NDH)^2,4^, a high risk state for Type 2 diabetes (T2D)^5,6^ also referred to as pre-diabetes or impaired glucose regulation. The scale of this problem has led to the introduction of population-based screening programmes for diabetes in some countries^7^. However, evidence supporting the widespread introduction of such programmes is mixed^7–13^. Randomised clinical trials have not demonstrated improvements attributable to diabetes screening on long-term health outcomes including microvascular and macrovascular complications and mortality^14–16^. While modelling studies indicate some long-term benefit from screening^17,18^, predictions are highly sensitive to the underlying assumptions and these may not reflect real-world conditions^19^. This lack of evidence has led many government and professional organisations, including the UK’s National Screening Committee, to advise against systematic population-based screening^16,20^.

**Figure 1:**
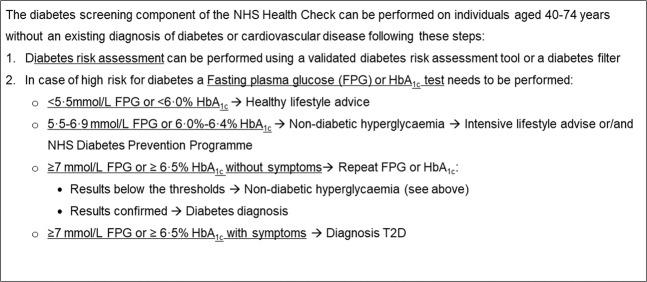
The diabetes risk assessment and screening component of the NHS Health Check Programme. In case of impossibility to use a validated diabetes risk assessment tool, a diabetes filter can be used. In this case, individuals at high risk of diabetes are identified as: (i) individuals from Black, Asian and other ethnic groups with BMI greater than 27.5 (ii) individuals with BMI greater than 30 (iii) individuals with blood pressure at or above 140/90 mmHg.

The NHS Health Check programme, launched in 2009 in England and rolled out nationally, is one of the world’s largest cardiovascular risk assessment and management programmes. Although its primary focus is cardiovascular risk assessment, the programme also includes a diabetes risk assessment and screening component (Fig. 1)^21^. The whole Health Check eligible population undergoes a T2D risk assessment and for those at increased risk of T2D a test to measure blood glucose level is offered (Fig. 1)^21^. A strong political commitment to the programme within the context of delivery within a health system with universal coverage, and with well-developed primary care and high penetration of electronic health records, presents an important opportunity to determine whether population-based screening for diabetes produces health benefit in real world settings. To date, no previous studies have reported the evaluation of the diabetes screening component of the Health Check programme.

This retrospective cohort study aims to determine whether the NHS Health Check programme increased the detection of T2D and NDH, and improved control of blood glucose and cardiovascular risk factors among newly diagnosed cases. Coverage of the NHS Health Check programme within general practice (defined for each general practice as the number of programme attendees divided by the total number of individuals registered with the practice who are eligible to attend the programme, Fig. 1) was considered as exposure. We consider this approach superior to directly comparing outcomes between Health Checks attendees and non-attendees for several reasons. Firstly, the programme is largely delivered through general practices but there have been substantial variations in implementation (0–73% programme coverage in 2013)^22,23^, due to administration of the programme at a local level, differences in the characteristics of practice populations^23^ and ongoing controversy about its effectiveness. Secondly, programme coverage is a proxy of general practices’ behaviour towards prevention. General practices’ active engagement in the NHS Health Check programme may be associated with increased opportunistic screening outside of the programme that would not be captured by comparing attendees and non-attendees. This is supported by the finding of a national evaluation of the NHS Health Check that showed a strong underlying trend of improvements in the testing and management of CVD risk factors among those who did not attend the programme^24^. Thirdly, similar analytical approaches are often used in policy evaluations as well as clinical trials (i.e. intention-to-screen analysis) to quantify benefits among people targeted by the intervention irrespective of their actual participation, providing an estimate of the effectiveness of the intervention^25^.

## Results

Coverage of the Health Check programme between 2009 and 2011 ranged from 0.5 to 61.6% with median values (interquartile range) of: (i) 8.5% (6.3 to 10.2%); (ii) 15.4% (13.6 to 17.5%); and (iii) 26.3% (22.8 to 34.6%) among low, medium, and high programme coverage practices, respectively. The three groups were largely similar in terms of sex and mean age at baseline, while they differed in ethnicity, smoking status, BMI, and prescription of anti-hypertensive treatment (Table 1). At baseline, the mean diabetes risk score was 5.9 in the total study population with 17.7% of the individuals being at high risk of T2D (DRS ≥ 10). Mean DRS and percentage of individuals at high risk of T2D varied significantly between groups (Table 1; Supplementary Table S1).

**Table 1:** Characteristics of study population at baseline according to the general practices’ coverage of the NHS Health Check programme and individuals’ diabetes risk score.

	Low Coverage*		Medium Coverage*		High Coverage*		Total		P VALUE
	DRS ≥ 10	TOTAL	DRS ≥ 10	TOTAL	DRS ≥ 10	TOTAL	DRS ≥ 10	TOTAL	
**N**	22860 17.4%	131160 100.0%	21624 17.5%	123716 100.0%	17299 18.4%	94111 100.0%	61783 17.7%	348987 100.0%	
**GENDER**									
Female	39.8%	49.8%	41.4%	49.8%	42.4%	49.8%	41.1%	49.7%	0.849
**AGE (years)**	58.7 (9.8)	50.4 (10.7)	58.1 (10.3)	49.9 (10.6)	57.8 (9.2)	49.8 (8.7)	58.2 (9.9)	50.1 (10.6)	p < 0.001
**ETHNICITY**									p < 0.001
White	50.1%	47.5%	52.1%	48.7%	60.0%	54.4%	54.6%	49.8%	
Non white	2.3%	3.7%	4.8%	7.3%	5.9%	8.8%	4.2%	6.4%	
Missing ethnicity	47.6%	48.8%	43.1%	44.0%	34.1%	36.8%	42.2%	43.9%	
**PRACTICE IMD**									p < 0.001
1Q – least deprived	17.5%	20.0%	17.4%	22.2%	11.0%	12.7%	15.6%	18.8%	
2Q	22.1%	23.8%	19.9%	20.7%	19.2%	20.5%	20.5%	21.8%	
3Q	28.7%	28.1%	21.0%	20.9%	13.8%	14.4%	21.8%	21.8%	
4Q	19.2%	17.5%	22.7%	20.3%	24.1%	23.7%	21.8%	20.2%	
5Q – most deprived	12.4%	10.6%	19.0%	15.9%	31.9%	28.7%	20.2%	17.4%	
**SMOKING STATUS**									p < 0.001
Non-smoker	50.9%	60.0%	48.8%	59.0%	46.6%	56.0%	48.9%	58.6%	
Ex-smoker	28.00%	18.1%	27.3%	17.4%	27.0%	17.5%	27.5%	17.7%	
Current smoker	21.16%	21.9%	23.9%	23.6%	26.4%	26.5%	23.6%	23.7%	
**BMI (kg/m**^**2**^**)**	32.6 (5.2)	27.0 (6.5)	32.6 (5.4)	27.1 (6.9)	32.7 (5.4)	27.3 (5.7)	32.8 (5.9)	27.1 (5.9)	p<0.001
**ANTHYPERTENSIVE TREATMENT**	36.5%	11.6%	34.6%	11.0%	36.7%	12.3%	35.8%	15.6%	p < 0.001
**DIABETES RISK SCORE**	17.3 (8.6)	5.8 (7.2)	17.5 (9.6)	5.8 (7.0)	17.9 (8.7)	6.2 (7.7)	17.6 (9.1)	5.9 (8.2)	p < 0.001
**T2D diagnoses before Jan 2009**^**§**^		5,352 3.9%		5,658 4.4%		4,926 5.0%		15,936 4.4%	

Results are reported as proportion for categorical variables and mean and standard deviation for continuous variables. Baseline differences between individuals registered with a practice with low, medium, and high programme coverage (‘total column’ for each group) were tested using Chi-square, T-test, and analysis of covariance, as appropriate. Results are shown as p-value in the last column. Legend: *Tertiles of general practices’ coverage of the NHS Health Check Programme were defined based on programme coverage during the first three years after its implementation (2009–11);

^§^Individuals with Type 2 Diabetes diagnosed before 1 Jan 2009 were not included in the analysis. Abbreviation: DRS = Diabetes Risk Score; T2D = Type 2 Diabetes.

### Incident cases of NDH and T2D

Mean follow-up of the entire study population was 7.8 ± 0.9 years, while for those at high risk of T2D at baseline mean follow-up was 6.8 ± 1.6 years. Over the study period, frequency of glycaemic testing was higher for patients registered with high coverage practices (low coverage practices: 54.9% of eligible population tested, mean number of tests per person 1.5 (2.4); high coverage practices: 66.7% of eligible population tested, mean number of tests per person 2.1 (2.9); Supplementary Table S2). 0.8% of the study population met the diagnostic criteria for NDH at baseline, while 7,126 cases (2.3%) were detected during the study period corresponding to an incidence of newly detected NDH of 0.23 per 1000 person-years. Incidence rates were 15% and19% higher for the medium and high coverage practices, respectively (medium coverage practices: HR 1.15, 95%CI (1.08–1.22); high coverage practices: 1.19 (1.11–1.27)), compared with the low coverage practices. Among patients at high risk of T2D at baseline, incidence rate of newly detected NDH in high coverage practices was 23% higher than in low coverage practices (HR 1.23 (1.11–1.37)).

**Figure 2:**
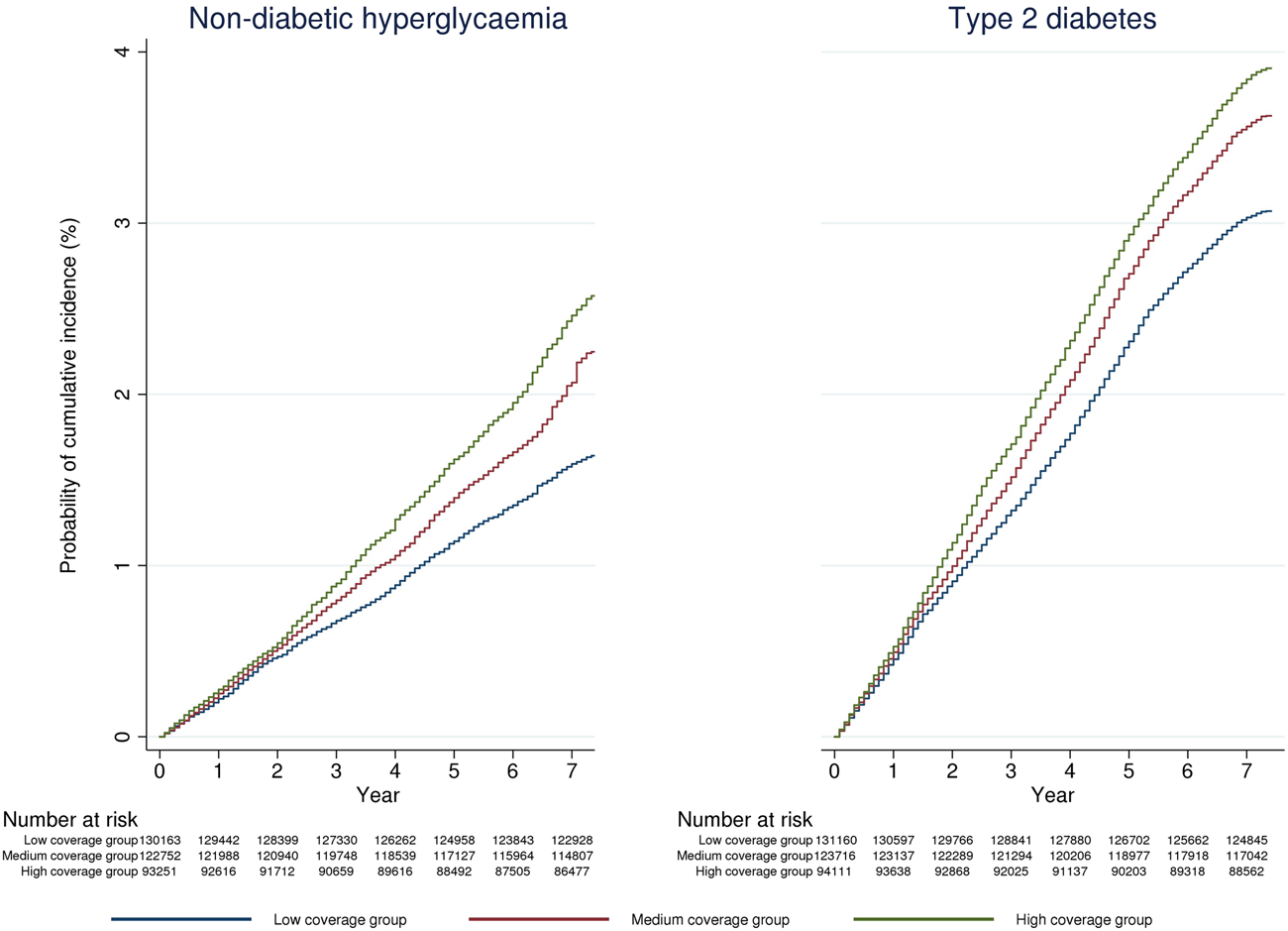
Kaplan Meier curves showing estimated rates of newly detected non-diabetic hyperglycaemia and newly diagnosed Type 2 Diabetes between Jan 2009 and May 2016 by general practices’ coverage of the NHS Health Check. In the calculation of individuals at risk of being detected with non-diabetic-hyperglycaemia (NDH), those meeting diagnostic criteria for NDH before 2009 were excluded at baseline and those with incident type 2 diabetes were progressively excluded.

Over the study period, 12,171 new cases of T2D were diagnosed, corresponding to an incident rate of 0.64 per 1000 person-years. Incident rates were 10% and 9% higher in the medium and high coverage practices respectively (medium coverage practices: 1.10 (1.05–1.15); high coverage practices: 1.09 (1.03–1.14), compared with low coverage practices. Further increase in rates of new T2D diagnoses was evident when restricting the analyses to individuals at high risk of diabetes (Fig. 2, Table 2).

**Table 2:** Differences in incidence rates of diagnoses of non-diabetic hyperglycaemia and type 2 diabetes by general practices’ coverage of the NHS Health Check Programme and patients’ baseline diabetes risk score.

	Incidence Rate	Total Sample			DRS ≥ 10		
		HR	95% CI		HR	95% CI	
**Non-Diabetic Hyperglycaemia**							
**Programme Coverage**^**§**^							
Low	0.19 per 1000 person-years (N = 2,096)	ref			ref		
Medium	0.25 per 1000 person-years (N = 2,693)	1.15***	1.08	1.22	1.15**	1.04	1.27
High	0.29 per 1000 person-years (N = 2,337)	1.19***	1.11	1.27	1.23***	1.11	1.37
**Type 2 Diabetes**	**Crude Rates**	**HR**	**95% CI**		**HR**	**95% CI**	
**Programme Coverage**^**§**^							
Low	0.35 per 1000 person-years (N = 4,018)	ref			ref		
Medium	0.42 per 1000 person-years (N = 4,482)	1.10***	1.05	1.15	1.13**	1.05	1.21
High	0.45 per 1000 person-years (N = 3,671)	1.09***	1.03	1.14	1.10*	1.02	1.19

Time period was Jan 2009-May 2016. Results are shown from multivariable Cox regression models. All models have been adjusted for the baseline values of the following independent variables: age, gender, ethnicity, smoking status, body mass index, antihypertensive medication, general practice deprivation score, and region. Models have also been adjusted for propensity score based on patients’ probability of being registered with a practice with low, medium or high coverage of the Health Check Programme. Abbreviations: DRS = diabetes risk score, HR = Hazard ratio. Legends: *p < 0.05, **p < 0.01, ***p < 0.001, ^§^Tertiles of general practices coverage of the NHS Health Check Programme were defined based on programme coverage during the first three years after its implementation (2009–11).

### Management of blood glucose

Compared with individuals at high risk of T2D registered with low coverage practices, those at high risk registered with high coverage practice had 0.1 mmol/L lower mean FPG and 48% greater likelihood of having blood glucose levels below the diagnostic criteria for NDH (FPG: β coefficient (95% CI) −0.09 (−0.12, −0.05); blood glucose levels below diagnostic criteria: OR 1.48 (1.43, 1.53)), over the study period.

**Figure 3:**
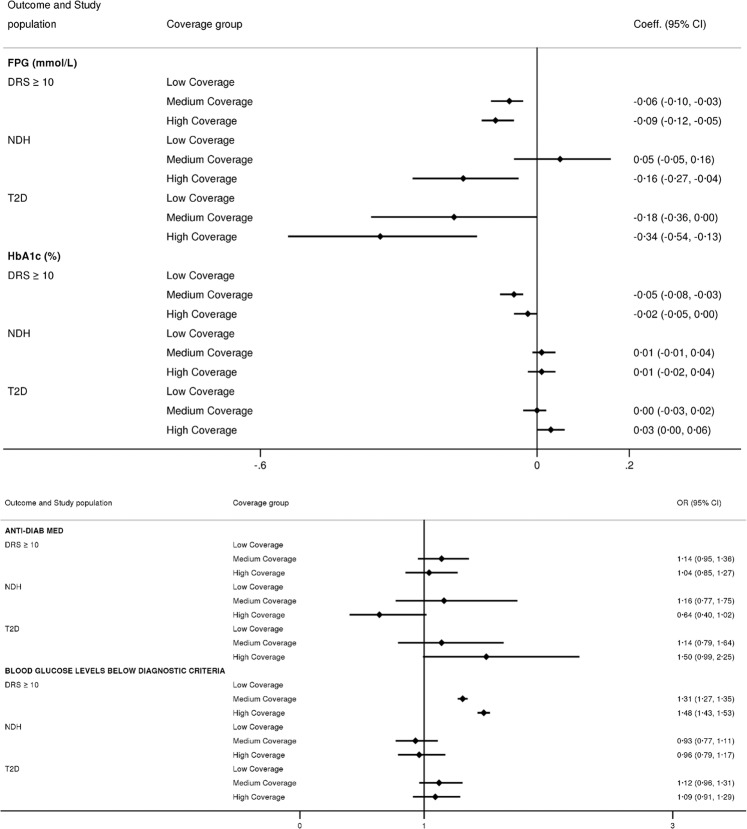
Differences in fasting plasma glucose levels and prescription of anti-diabetic medications according to general practices’ coverage of the Health Check programme, patients’ baseline diabetes risk score, and new diagnoses of non-diabetic hyperglycaemia and type 2 diabetes between 2009 and 2016 in England. Time period was from 1 January 2009 to 31 May 2016. Tertiles of general practices coverage of the NHS Health Check Programme were defined based on programme coverage during the first three years after its implementation (2009–11). Results are shown from mixed-effect linear regression models for continuous outcomes and mixed-effect logistic regression models for binary outcomes. In both models ‘Low Coverage’ group has been used as referent category. Independent variables included in the model are the following: practices’ early programme coverage of the NHS Health Check programme, year, baseline age, gender, ethnicity, smoking status, BMI, antihypertensive medication, presence of cardiovascular disease, general practice IMD, and region. Models have also been adjusted for propensity score based on patients’ probability of being registered with a practice with low, medium or high coverage of the Health Check Programme. Abbreviations: DRS = diabetes risk score, NDH = non-diabetic hyperglycaemia, OR = Odds Ratio.

Among incident NDH cases, those registered with high coverage practices had a 0.2 mmol/L lower mean FPG (−0.16 (−0.27–−0.04)) over the follow-up period compared with patients registered with low coverage practices. Among individuals with incident T2D, those registered with high coverage practice had a 0.3 mmol/L lower mean FPG (−0.34 (−0.54–−0.13). Detailed results are presented in Fig. 3.

No differences were found between groups for the likelihood of receiving anti-diabetic medications among individuals with incident T2D following the diagnosis.

### Cardiovascular risk factor management

#### Blood pressure

**Figure 4:**
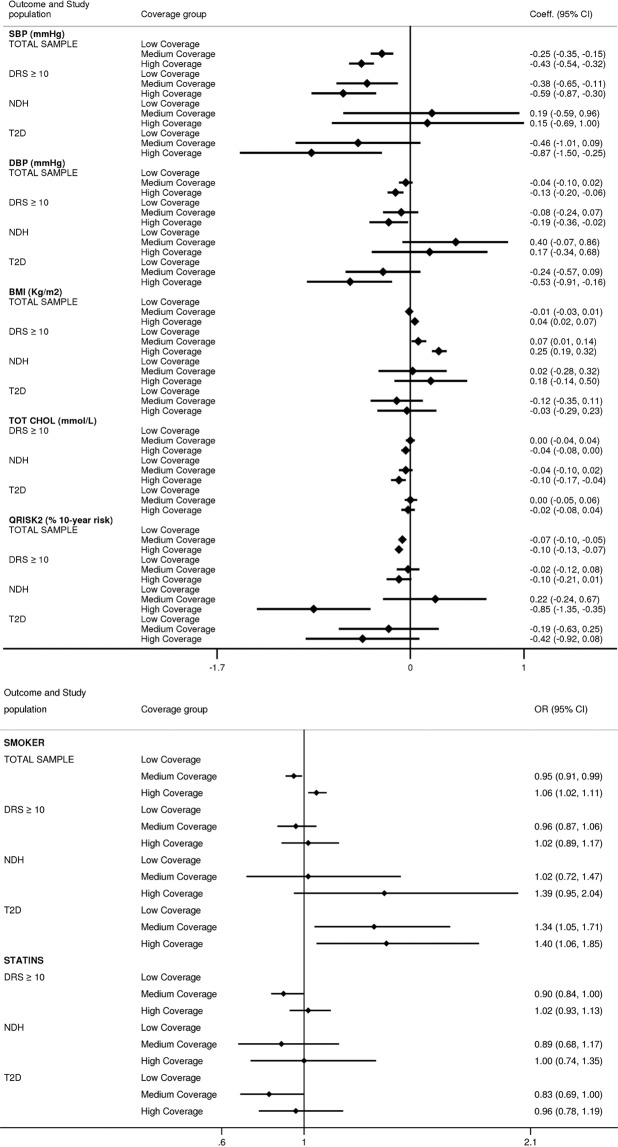
Differences in cardiovascular risk factors between 2009 and 2016 by general practices’ coverage of the NHS Health Check programme and individuals’ baseline diabetes risk. Time period was from 1 January 2009 to 31 May 2016. Tertiles of general practices coverage of the NHS Health Check Programme were defined based on programme coverage during the first three years after its implementation (2009–11). Results are shown from mixed-effect linear regression models for continuous outcomes and mixed-effect logistic regression models for binary outcomes. In both models ‘Low Coverage’ group has been used as referent category. Independent variables included in the model are the following: practices’ early programme coverage of the NHS Health Check programme, year, baseline age, gender, ethnicity, smoking status, BMI, antihypertensive medication, presence of cardiovascular disease, general practice IMD, and region. To calculate estimates for individuals with a DRS ≥ 10 an interaction term between early programme coverage and DRS has also been included. Differences in total cholesterol levels and statins prescription have been restricted to only those with a DRS ≥ 10 at baseline. Models have also been adjusted for propensity score based on patients’ probability of being registered with a practice with low, medium or high coverage of the Health Check Programme. Abbreviations: DRS = diabetes risk score, NDH = non-diabetic hyperglycaemia, T2D = type 2 diabetes, OR = Odds Ratio.

Over the study period, compared with those registered with a low coverage practice, individuals registered with medium and high coverage practices had a mean SBP 0.3 and 0.4 mm Hg lower (β coefficient (95% CI) −0.25 (−0.35–−0.15); −0.43 (−0.54–−0.32), repectively). Difference was more pronounced among individuals at high risk of T2D, with a 0.4 and 0.6 mm Hg lower mean in the medium and high coverage tertiles (medium coverage practices:– 0.38 (−0.65–−0.11); high coverage practices: −0.59 (−0.87–−0.30)), respectively. Individuals with newly diagnosed T2D registered with high coverage practices had 0.9 mmHg lower SBP mean (−0.87 (−1.50–−0.25)). Results for the mean DBP were qualitatively similar (Fig. 4).

#### Body mass index

Among those at high risk of T2D, individuals registered with high coverage practices had 0.3 kg/m^2^ higher mean BMI (β coefficient (95% CI) 0.25 (0.19–0.32)), compared with the low coverage group. No differences were found at follow-up among individuals meeting the diagnostic criteria for NDH and with newly diagnosed T2D (Fig. 4).

#### Smoking prevalence

Individuals registered with a high coverage practice had 6% greater likelihood of being smoker (OR (95% CI) 1.06 (1.02–1.11)), compared with low coverage practices. For incident cases of T2D the likelihood of being smoker over the follow-up period was 34% and 40% greater for those registered with medium and high coverage practices, compared with those registered with low coverage practices (Fig. 4).

#### Total cholesterol

Individuals with incident NDH registered with high coverage practices had a 0.1 mmol/L lower mean total cholesterol (β coefficient (95% CI) −0.10 (−0.17–−0.04)), compared with those registered with low coverage practices (Fig. 4). No difference between the two groups were found in prescribing of statins following the detection of NDH.

#### Modelled CVD risk

Compared with those registered with low coverage practices, individuals registered with medium and high coverage practices had a 0.1% lower modelled CVD risk score. Differences were more pronounced among individuals meeting diagnostic criteria for NDH which, on average, had a 0.9% lower cardiovascular risk score after the detection (β coefficient (95% CI −0.85 (−1.35–−0.35)).

### Sensitivity analyses

Results of the regression analyses without propensity score adjustment were largely similar to the main findings and are presented in Supplementary Tables S3–S6.

## Discussion

In this retrospective analysis of prospectively collected data using a representative sample of the English population, we assessed the impact of the diabetes risk assessment and screening component of the NHS Health Check programme on the detection of NDH and T2D and levels of blood glucose and cardiovascular risk factors among individuals at high risk of T2D and newly diagnosed cases. We found that general practices with the highest programme coverage had 19% and 9% higher detection rates of NDH and T2D respectively, compared with practices with the lowest coverage. Compared with counterparts registered with low coverage practices, individuals with incident NDH registered with high coverage practices had lower levels of fasting plasma glucose and cholesterol over the study period but similar blood pressure and BMI levels. Individuals newly diagnosed with T2D in high coverage practices had lower levels of blood pressure, and fasting plasma glucose, but similar levels of cholesterol, and BMI. Furthermore, individuals with NDH registered with practices with higher coverage had a 0.9% lower modelled 10-year cardiovascular risk score compared with individuals in low coverage practices.

The clinical benefits of large-scale programmes directed at the identification and management of individuals at high risk of diabetes remain unclear^7,10–13^. Evidence from randomised trials suggests that early detection of abnormal glucose metabolism through screening and subsequent intensive management reduce cardiovascular risk among individuals with NDH and newly diagnosed T2D^11,26^. The results of the ADDITION-Denmark study suggest that diabetes screening was associated with a significant reduction in risk of all-cause mortality and CVD events in those diagnosed with diabetes^27^. However, criticisms have been raised on the interpretation of these findings due to selection of non-randomised control population for the study^12,13^.

Results on the benefit of diabetes screening in reducing CVD risk were also confirmed by a modelling study using data from randomised trials^11^. Although it is difficult to make direct comparisons with other studies due to differences in design and settings, our results also indicate better cardiovascular risk factor control in individuals with newly detected NDH and newly diagnosed T2D who were registered with practices that more widely adopted diabetes screening as part of Health Check. Our findings also suggest that the intensity of control of cardiovascular risk factors is aligned with increased clinical risk, and this appears to be more evident among practices that adopt systematic risk assessment and management strategies.

Similarly, individuals at high risk of T2D, and patients with newly detected NDH and T2D registered with high coverage practices had lower fasting plasma glucose levels than those registered with low coverage practices. This finding is also in line with previous studies reporting lower blood glucose levels among individuals with screen-detected T2D compared with patients detected in routine clinical care^28^.

### Strengths and limitations

Data on the impact of diabetes risk assessment and screening programmes in real world settings are scarce. The NHS Health Check is one of the largest such programmes globally and its delivery in a universal health system with high penetration of electronic health records provides a unique opportunity for evaluation. We employed a robust study design using a representative sample of the English population^29^. We used general practice coverage of the Health Check programme as our exposure, which arguably captures whether practices adopt a pro-active approach to prevention, including identifying individuals at high risk of T2D. This design may also reduce possible selection bias, such as the ‘healthy screenee bias’, that might occur when directly comparing those who attend and those who do not attend a screening programme. Our findings, therefore, cannot be directly attributable to programme attendance because they also capture differences between practices in their approach to pro-actively perform more opportunistic screenings as well as better embed national guidelines into clinical practice.

Several caveats merit discussion. In line with what we found, it has been reported that the coverage of the NHS Health Check in the first years of the programme was low, with considerable practice-level variations^23^. In England, variations in the coverage of national programmes^22,23^ have been attributed to differences in the organisation of general practices, socio-economic deprivation and patient health status and preferences^22,23,30^. In the present study the three coverage groups differed in socio-demographic and clinical characteristics at baseline, and we sought to reduce this possible source of bias by adjusting analyses for the propensity of being registered with a general practice with a specific level of programme coverage. Although the analysis only included individuals without cardiovascular disease and diabetes at baseline, we reported baseline diabetes prevalence across tertiles of programme coverage, which was higher in the high coverage group. Similarly, for individuals included in the analysis the baseline DRS was slightly higher in the high coverage group as compared with the low coverage. This might partially attenuate our findings, which might also reflect proportionality in diabetes risk and greater underlying diabetes incidence in high coverage groups. Other limitations include missing values at baseline for blood pressure and BMI records, variables required for the estimation of baseline risk of T2D and propensity scores. We addressed this by using multiple imputation and including a wide range of clinical and socio-demographic variables that may be predictive of the missing data. We conducted complete-case analyses for the FPG, HbA1c, and total cholesterol study outcomes due to presence of missing data. Additionally, over the study period there were changes in the cardiovascular and diabetes process indicators selected as part of the UK Quality and Outcome Framework pay-for-perfomance programme (e.g. 5 mmol/L target for total cholesterol in individuals with T2D was retired as process indicator during the study period) and this might have partially influenced our findings. Finally, when using routinely collected data concerns have been raised about miscoding, misclassification and misdiagnosis. However, CPRD is a reliable widely used data source and is subject to regular quality checks^31^.

### Policy implications

The English National Health Service has invested considerable resources in improving the early detection and management of diabetes through the NHS Health Checks and the recently introduced NHS Diabetes Prevention Programme, which involves intensive lifestyle interventions among CVD risk management in individuals with NDH. Our findings show that general practices that actively participated in the NHS Health Check programme had not only tested a greater proportion of the eligible population and detected larger number of previously undiagnosed NDH and T2D cases but achieved better glucose and cardiovascular risk management among individuals identified with high T2D risk and newly diagnosed T2D. This is particularly important, considering the currently existing variations in programme delivery across general practices regarding coverage and uptake of interventions offered through the programme that have the potential to improve health. These findings are encouraging given that patients with NDH or T2D have an increased risk for cardiovascular morbidity and mortality. However, the long-term effects of diabetes risk assessment programmes on hard clinical outcomes and the burden of T2D warrant further research. Furthermore, it is still unclear at what scale would diabetes risk assessment programmes generate population-level impacts while remaining cost-effective. Such person-centred interventions require a higher level of engagement from individuals and their impact on health inequalities require rigorous evaluation. Besides focusing on early detection of diabetes and assessment of individuals at high risk, it is important that policy interventions include approaches which reduce T2D risk factors across the entire population, regardless of person-level risk.

## Conclusions

We found that general practices’ actively participating in the NHS Health Check programme had higher detection of NDH and T2D and better management of cardiovascular disease risk in newly diagnosed cases. However, findings might be partially attenuated by a greater underlying diabetes incidence in general practices with higher programme coverage. Further evaluation is required on long-term population-level health impacts and cost-effectiveness combined with information on effects on health inequalities before widespread implementation of similar programmes can be recommended, especially in settings with limited healthcare resources.

## Methods

### Study design

This is a retrospective analysis of prospectively collected data that compared selected health outcomes of individuals registered with general practices with different levels of programme coverage (tertiles). Analyses were controlled for individuals’ likelihood of accessing the programme using propensity score regression adjustment. Individuals were included in the analyses irrespective of their participation in the programme, similarly to an intention-to-screen design^14^.

### Data source

We used data from the Clinical Practice Research Datalink (CPRD), one of the largest databases of electronic medical records in the world^31^. CPRD routinely collects longitudinal and anonymized primary care data from participating general practices in the United Kingdom (UK), covering approximately 7% of the UK population and it is representative in terms of age and sex^31^. CPRD provides linkage to hospital admission and mortality data^31^. Currently 75% of CPRD practices in England have consented to linkage^32^. Data are subject to regular quality checks and are widely used for research studies^31,33^. CPRD has been widely used in the evaluation of the NHS Health Check programme^24^ and other national strategies implemented in a primary care setting like the Quality and Outcomes Framework^34–36^.

### Ethical approval

CPRD has been granted Multiple Research Ethics Committee approval (05/MRE04/87) to undertake purely observational studies with external data linkages, including hospital admission and mortality data. The present study is based on anonymised and unidentifiable CPRD data. Ethical approval for the study protocol was granted by the Independent Scientific Advisory Committee of the CPRD (protocol number: 15_250R). All the study methods were performed in accordance with the relevant guidelines and regulations and in accordance with best scientific practice.

### Study population

We obtained data for a computer-selected random sample of 387,460 individuals aged 40–74 years who were continuously registered with 455 CPRD general practices in England between 1 January 2009 and 31 December 2014 and whose data were deemed up-to-standard by the CPRD quality-check. After excluding individuals with a diagnosis of CVD and T2D before 1 January 2009, 348,987 individuals eligible for the NHS Health Check programme were included in this study (Supplementary Fig. S1). To allow a longer follow-up of our study population, we obtained an update of CPRD data that capture study outcomes up to 31 May 2016.

### NHS health check programme coverage

We defined practice-level Health Check coverage during the first three years of the programme (2009–2011) as the number of attendees divided by the number of Health Check eligible individuals registered with the practice. We identified Health Check attendance using an established algorithm^23,24^ because the coding system in general practice electronic records for Health Check attendance was poorly implemented during early phase of the programme^23^. In line with the NHS Best Practice Guidance, the programme attendance includes the measurement of four risk factors: blood pressure, body mass index, serum cholesterol and smoking status, recorded within a six-month window during the intervention period. The published algorithm was developed based on this definition with the attendance date assigned as the date when the last of the four risk factors was recorded^23^. We considered programme coverage in the first three years as the exposure to allow sufficient follow-up time to detect changes in our outcome measures following programme implementation. We divided general practices into tertiles based on their Health Check coverage during the first three years of the programme. Individuals were therefore categorised into 3 groups according to their registered practices’ tertile of Health Check coverage.

### Diabetes risk score

We used ‘QDiabetes’^37^, a validated diabetes risk assessment tool^38^ to compute individuals’ diabetes risk score (DRS) on the 1^st^ January 2009. Individuals with a DRS ≥ 10 were considered at high risk of T2D^37,38^.

### Outcomes

The diagnosis of NDH was based on Read codes (Supplementary Table S7) or laboratory blood tests following the WHO diagnostic laboratory criteria for NDH (fasting plasma glucose (FPG): 6.1–6.9 mmol (111 mg/dl and 125 mg/dl) or glucose levels 2 hours after oral glucose tolerance test (OGTT) 7.8–11.1 mmol/L or glycated haemoglobin (HbA1c): 42 to 47 mmol/mol (6.0–6.4%)). New T2D diagnoses were determined using both primary care (Read codes) and hospital admission records data (International Statistical Classification of Diseases and Related Health Problems, 10th Revision (ICD-10) codes), as previously recommended^39^. Only a small proportion (3%) of individuals at high risk of T2D at baseline had data on OGTT recorded, therefore, we present FPG and HbA1c as glycaemic outcome measures in this study.

Data on study outcomes obtained for each cohort year included systolic blood pressure (SBP), diastolic blood pressure (DBP), body mass index (BMI), smoking status, and 10-year modelled CVD risk score. Smoking status was defined as smoker, non-smoker or ex-smoker. CVD risk score was estimated based on the ‘QRISK2’ algorithm, as recommended by the National Institute for Health and Care Excellence^40^.

Data on FPG, HbA1c, total cholesterol and prescription of oral anti-diabetic medications and statins, were obtained for each cohort year for people at high risk of T2D at baseline, and for individuals with newly detected T2D and NDH for each year following detection. Given that the use of HbA1c was introduced as a diagnostic test in UK national clinical guidelines in 2012, FPG was more widely available in the study, with a high proportion of missing data for HbA1c^37^. Therefore, we constructed an additional binary variable for a combined measure of blood glucose as follows: NDH: FPG mmol/L 6.1–6.9 mmol/L or HbA1c 42 to 47 mmol/mol (6.0–6.4%; T2D: FPG ≥ 7.0 mmol/L or HbA1c ≥ 48 mmol/m (6.5%))^37^. The binary variable was coded as ‘1’ if blood glucose levels were below the clinical criteria to define NDH or T2D (e.g. for individuals at high risk of T2D and with incident NDH: FPG < 6.1 mmol/L and HbA1c < 42 mmol/m; for those with incident T2D: FPG < 7.0 mmol/L and HbA1c < 48 mmol/m).

### Study covariates

Study covariates included practice and patient characteristics. Practice characteristics included English geographic region, index of multiple deprivation (IMD), and case-load (defined as the number of individuals eligible for the Health Check programme within the practice). Individual characteristics included age, sex, ethnicity (white, non-white or missing), number of co-morbidities (using a previously published list of comorbidities^41^), and prescription of anti-hypertensive, antidiabetic, lipid lowering and steroid medications.

### Statistical analysis

We aimed to compare study outcomes between individuals registered with general practices with different levels of participation (defined as the tertiles of practice coverage of eligible individuals) in the Health Check programme. We adopted a propensity score regression adjustment in order to reduce selection bias^42^. We estimated three sets of propensity scores using logistic regression models. Each model generated propensity scores based on the probability of individuals being registered with a practice with a specific level of programme coverage compared with the other groups (low coverage vs. medium coverage; low coverage vs. high coverage; medium coverage vs. high coverage). All logistic regression models were adjusted for baseline variables that may be associated with programme coverage, this included age, sex, ethnicity, BMI, SBP and DBP, IMD, case-load, and geographical region.

To reduce individuals’ missing data at baseline, we used the latest clinical data for each individual within 5 years before the start of the study period^24^. We then used multiple imputation by chained equations (10 copies) to estimate missing data for BMI and blood pressure at baseline because these variables were needed to calculate the baseline DRS and the propensity scores. We included the following covariates in the imputation model: age, gender, ethnicity, smoking status, number of co-morbidities, anti-hypertensive medication, lipid-lowering medication, steroid medication, practice IMD, and geographical region. Estimates were combined using Rubin’s rule. Individuals with missing data on smoking were classified as non-smokers if there was no indication in the past of the patient being a smoker^43^.

We assessed the unadjusted differences in the population characteristics between individuals registered with low, medium and high programme coverage practices using Chi-square, t-test and ANOVA, as appropriate. Cox Proportional Hazards regression models were used to estimate the hazard ratios for the detection of NDH and T2D among the three groups, using the low coverage group as reference. These analyses were conducted on different sub-populations: (i) total sample (ii) individuals at high risk of T2D (DRS at baseline ≥10). Individuals were considered at risk for the entire study period or until reaching study endpoints and censored in case of death, transfer out of the general practice or end of study period. These models were adjusted for baseline covariates including a combination of both practice (region and IMD) and individual (age, gender, ethnicity, smoking status, BMI, and anti-hypertensive medication) characteristics. All models were further adjusted for the three propensity scores generated. The assumption of parallel hazard functions over time was met. To test this assumption we examined plots of log(−log survival time) against log survival time and Schoenfeld residuals against survival time. In addition, we used linear regression of Schoenfeld residuals on time to test for independence between residuals and time. We examined whether the programme was associated with improved risk factor control by comparing individuals’ outcome data between low, medium, high programme coverage groups during the study period (1 January 2009–31 May 2016). We used two-level mixed-effect regression models (repeated measures within each individual) to account for the hierarchical structure of the data when computing standard errors. Specifically, we used mixed-effects linear regression models for continuous outcomes and mixed-effects logistic regression models for binary outcomes. For continuous outcomes, including FPG, HbA1c, SBP, DBP, BMI, total cholesterol, and QRISK2, we calculated the annual mean value of the outcome in case of multiple measurements within a year for each individual. For binary outcomes including blood glucose targets, smoking status and prescription of antidiabetic medication, we considered the latest data recorded within a year for each individual. The mixed-effect analyses were conducted on different sub-populations: (i) total sample (ii) individuals at high risk of T2D (DRS at baseline ≥10) (iii) individuals with newly detected NDH iv) individuals with newly diagnosed T2D. For the third and fourth models, the time period was defined as the time between the year of diagnosis and the end of the study period (31 May 2016). Analyses on FPG, HbA1c, and total cholesterol were only performed on individuals at high risk of T2D and with newly detected NDH and T2D because monitoring of these parameters is only recommended by national guidance for people at high risk of T2D or diagnosed T2D^37^. Similarly, prescription of anti-diabetic medications and statins were only analysed in sub-populations 2, 3, and 4 in order to assess differences in pharmaceutical approach in individuals at high risk or with incident NDH or T2D. Assumptions of the mixed-effect linear regression models were tested graphically for the violations against normality of random effects and homogeneity of residual variance. No evidence of violations against assumptions and no apparent outliers were identified. These models were adjusted for year (of the outcome recorded), and for baseline covariates including a combination of both practice (region and IMD) and individual (age, gender, ethnicity, smoking status, BMI, and anti-hypertensive medication) characteristics. All models were further adjusted for the three propensity scores generated.

Sensitivity analyses were performed using regression models without adjustment for propensity scores. Considering the percentage of missing data for the FPG, HbA1c, and total cholesterol outcomes (13.6% for total cholesterol, 28.5% for fasting plasma glucose, and 67.6% for HbA1c in individuals at high risk of T2D), we performed complete-case analysis. Percentages of missing data are reported in Supplementary Table S8.

## Supplementary information

Supplementary Material.

## Data Availability

The data that support the findings of this study are available from CPRD but restrictions apply to the availability of these data, which were used under license from the UK Medicines and Healthcare products Regulatory Agency for the current study, and so are not publicly available.
