# Supplementary material for: Evaluation of the Diabetes Screening Component of a National Cardiovascular Risk Assessment Programme in England: a Retrospective Cohort Study
Source: Sci Rep. 2020 Jan 27;10:1231. doi: 10.1038/s41598-020-58033-3 (PMC6985103; doi:10.1038/s41598-020-58033-3)
Supplement: Supplementary file 1 — Supplementary Material. [file 41598_2020_58033_MOESM1_ESM.docx]

**Supplementary Information**

Evaluation of the Diabetes Screening Component of a National Cardiovascular Risk Assessment Programme in England: a Retrospective Cohort Study.

Raffaele Palladino MD^1,2^, Eszter P Vamos PhD^1^, Kiara Chu-Mei Chang PhD^1^, Kamlesh Khunti PhD^3^, Azeem Majeed MD^4^, Christopher Millett PhD^1^

1. Public Health Policy Evaluation Unit, School of Public Health, Imperial College London, London, United Kingdom
2. Department of Public Health, “Federico II” University of Naples, Naples, Italy
3. Diabetes Research Centre, Leicester Diabetes Centre, University of Leicester, Leicester, United Kingdom
4. Department of Primary Care and Public Health, Imperial College London, London, United Kingdom

**Table S1.** Values of the study outcomes at baseline and follow-up by general practices’ coverage of the NHS Health Check programme and individuals’ baseline diabetes risk score.

|  | **TOTAL SAMPLE** | | **DRS ≥ 10** | |
| --- | --- | --- | --- | --- |
|  | **BASELINE VALUE** | **VALUE AT FOLLOW-UP^Ω^** | **BASELINE VALUE** | **VALUE AT FOLLOW-UP^Ω^** |
| **FASTING PLASMA GLUCOSE (mmol/L)** |  |  | |  |
| **PROGRAMME COVERAGE^§^** |  |  |  |  |
| **Low** |  |  | 5.5 (1.7) | 5.6 (1.7) |
| **Medium** |  |  | 5.4 (1.7) | 5.7 (1.7) |
| **High** |  |  | 5.4 (1.6) | 5.5 (1.5) |
| **HbA1c (%)** |  |  | |  |
| **PROGRAMME COVERAGE^§^** |  |  |  |  |
| **Low** |  |  | 4.6 (1.9) | 2.8 (0.5) |
| **Medium** |  |  | 4.3 (2.0) | 2.8 (0.4) |
| **High** |  |  | 4.4 (1.9) | 2.8 (0.4) |
| **ANTI-DIABETIC MEDICATIONS (%)** |  |  | |  |
| **PROGRAMME COVERAGE^§^** |  |  |  |  |
| **Low** |  |  | 1.0 | 4.6 |
| **Medium** |  |  | 1.2 | 5.8 |
| **High** |  |  | 1.2 | 6.0 |
| **SYSTOLIC BLOOD PRESSURE (mm Hg)** |  |  | |  |
| **PROGRAMME COVERAGE^§^** |  |  |  |  |
| **Low** | 129.9 (17.0) | 130.4 (15.5) | 138.0 mmHg (17.8) | 136.5 (14.7) |
| **Medium** | 129.6 (16.6) | 130.2 (15.5) | 137.4 mmHg (19.3) | 136.0 (15.0) |
| **High** | 129.7 (17.6) | 130.1 (15.2) | 137.0 mmHg (17.1) | 135.5 (14.6) |
| **DIASTOLIC BLOOD PRESSURE (mm Hg)** |  | **BASELINE VALUE (mmHg)** | |  |
| **PROGRAMME COVERAGE^§^** |  |  |  |  |
| **Low** | 79.1 (10.1) | 78.9 (9.4) | 82.3 (11.4) | 80.3 (9.3) |
| **Medium** | 79.1 (10.8) | 78.8 (9.6) | 82.1 (11.6) | 80.0 (9.5) |
| **High** | 79.1 (11.3) | 78.7 (9.4) | 82.0 (10.5) | 79.8 (9.3) |
| **BODY MASS INDEX (Kg/m^2^)** |  |  | |  |
| **PROGRAMME COVERAGE^§^** |  |  |  |  |
| **Low** | 27.2 (6.4) | 27.3 (5.4) | 32.6 (5.2) | 32.1 (5.6) |
| **Medium** | 27.2 (6.9) | 27.4 (5.5) | 32.6 (5.4) | 32.0 (5.8) |
| **High** | 27.4 (5.9) | 27.7 (5.5) | 32.7 (5.4) | 32.4 (5.7) |
| **SMOKING PREVALENCE (%)** |  |  | |  |
| **PROGRAMME COVERAGE^§^** |  |  |  |  |
| **Low** | 21.7 | 22.0 | 20.6 | 20.0 |
| **Medium** | 23.5 | 23.1 | 23.1 | 21.8 |
| **High** | 26.4 | 26.0 | 26.0 | 23.6 |
| **TOTAL CHOLESTEROL (mmol/L)^¥^** |  |  | |  |
| **PROGRAMME COVERAGE^§^** |  |  |  |  |
| **Low** |  |  | 5.2 (2.3) | 5.1 (1.8) |
| **Medium** |  |  | 5.2 (3.0) | 5.1 (2.7) |
| **High** |  |  | 5.1 (1.5) | 5.0 (1.3) |
| **STATINS (%)^¥^** |  | **BASELINE VALUE (%)** | |  |
| **PROGRAMME COVERAGE^§^** |  |  | |  |
| **Low** |  |  | 29.2 | 52.6 |
| **Medium** |  |  | 30.2 | 52.4 |
| **High** |  |  | 33.3 | 55.4 |
| **QRISK2 (% 10-year risk)** |  | **BASELINE VALUE (% 10-year risk)** | |  |
| **PROGRAMME COVERAGE^§^** |  |  | |  |
| **Low** | 6.1 (6.6) | 9.1 (9.5) | 9.9 (9.8) | 16.4 (11.9) |
| **Medium** | 5.8 (6.6) | 9.1 (9.8) | 9.4 (9.7) | 16.6 (12.6) |
| **High** | 5.9 (6.6) | 9.2 (9.8) | 9.4 (9.2) | 16.5 (12.5) |
|  |  |  |  |  |

§ Tertiles of general practices coverage of the NHS Health Check Programme were defined based on programme coverage during the first three years after its implementation (2009-11).

¥ Changes in the mean total cholesterol has only been assessed for individuals at high risk of T2D at baseline because national guidelines do not recommend cholesterol monitoring for the whole population.

**^Ω^** For each individual, the latest available value was considered.

**Table S2**. Intensity of plasma glucose testing by general practices’ coverage of the NHS Health Check programme

|  |  | **Any glycaemic test (FPG, HbA1c, OGTT)** | | **FPG** | | **HbA1c** | |
| --- | --- | --- | --- | --- | --- | --- | --- |
| **PROGRAMME COVERAGE^§^** | **N** | **% tested** | **Mean number of tests** | **% tested** | **Mean number of tests** | **% tested** | **Mean number of tests** |
| **Low** | **131,160** | **54.9** | **1.5 (2.4)** | **52.4** | **1.2 (1.8)** | **16.5** | **0.3 (1.0)** |
| **Medium** | **123,716** | **63.3** | **2.0 (2.8)** | **61.0** | **1.6 (2.0)** | **20.0** | **0.4 (1.1)** |
| **High** | **94,111** | **66.7** | **2.1 (2.9)** | **64.3** | **1.7 (2.1)** | **22.3** | **0.4 (1.2)** |

**RESULTS FROM SENSITIVITY ANALYSIS**

**Table S3.** Differences in incidence rates of diagnoses of non-diabetic hyperglycaemia and type 2 diabetes by general practices’ coverage of the NHS Health Check Programme and patients’ baseline diabetes risk score. Results obtained without adopting propensity score regression adjustment.

|  | **TOTAL SAMPLE** | | | **DRS ≥ 10** | | |
| --- | --- | --- | --- | --- | --- | --- |
|  | **HR** | **95% CI** | | **HR** | **95% CI** | |
| **NON-DIABETIC HYPERGLYCAEMIA** |  |  |  |  |  |  |
| **PROGRAMME COVERAGE^§^** |  |  |  |  |  |  |
| **Low** | Ref | | | ref | |  |
| **Medium** | 1.18*** | 1.11 | 1.25 | 1.19*** | 1.08 | 1.31 |
| **High** | 1.18*** | 1.11 | 1.26 | 1.22*** | 1.10 | 1.36 |
| **TYPE 2 DIABETES** | **HR** | **95% CI** | | **HR** | **95% CI** | |
| **PROGRAMME COVERAGE^§^** |  |  |  |  |  |  |
| **Low** | ref | | | ref | | |
| **Medium** | 1.12*** | 1.07 | 1.17 | 1.14*** | 1.06 | 1.23 |
| **High** | 1.09*** | 1.04 | 1.15 | 1.11* | 1.02 | 1.20 |

Note: Time period was Jan 2009-May 2016. Results are shown from multivariable Cox regression models. All models have been adjusted for the baseline values of the following independent variables: age, gender, ethnicity, smoking status, body mass index, antihypertensive medication, general practice deprivation score, and region. DRS = diabetes risk score, HR = Hazard ratio.

*p<0.05, **p<0.01, *** p<0.001.

§ Tertiles of general practices coverage of the NHS Health Check Programme were defined based on programme coverage during the first three years after its implementation (2009

**Table S4.** Differences in fasting plasma glucose levels and prescription of anti-diabetic medications according to general practices’ coverage of the Health Check programme, patients’ baseline diabetes risk score, and new diagnoses of non-diabetic hyperglycaemia and type 2 diabetes between 2009 and 2016 in England. Results obtained without adopting propensity score regression adjustment.

|  | **DRS ≥ 10** | | | **INCIDENT NON-DIABETIC HYPERGLYCAEMIA** | | | **INCIDENT TYPE 2 DIABETES** | | |
| --- | --- | --- | --- | --- | --- | --- | --- | --- | --- |
|  |  |  |  |  |  |  |  |  |  |
| **FASTING PLASMA GLUCOSE** | **Coeff.** | **95% CI** | | **Coeff.** | **95% CI** | | **Coeff.** | **95% CI** | |
| **PROGRAMME COVERAGE^§^** |  |  |  |  |  |  |  |  |  |
| **Low** | ref | | | ref | | | ref | | |
| **Medium** | -0.05** | -0.08 | -0.01 | 0.04 | -0.06 | 0.15 | -0.17 | -0.34 | 0.01 |
| **High** | -0.08*** | -0.11 | -0.04 | -0.18** | -0.29 | -0.06 | -0.37*** | -0.57 | -0.17 |
| **BLOOD GLUCOSE LEVELS BELOW DIAGNOSTIC CRITERIA** | **OR** | **95% CI** | | **OR** | **95% CI** | | **OR** | **95% CI** | |
| **PROGRAMME COVERAGE^§^** |  |  |  |  |  |  |  |  |  |
| **Low** | ref | | | ref | | | ref | | |
| **Medium** | 1.30*** | 1.26 | 1.33 | 0.91 | 0.76 | 1.1 | 1.11 | 0.95 | 1.29 |
| **High** | 1.47*** | 1.42 | 1.52 | 0.97 | 0.8 | 1.19 | 1.03 | 0.87 | 1.22 |
| **ANTI-DIABETIC MEDICATION** | **OR** | **95% CI** | | **OR** | **95% CI** | | **OR** | **95% CI** | |
| **PROGRAMME COVERAGE^§^** |  |  |  |  |  |  |  |  |  |
| **Low** | ref | | | ref | | | ref | | |
| **Medium** | 1.16 | 0.97 | 1.39 | 0.97 | 0.65 | 1.44 | 1.05 | 0.73 | 1.51 |
| **High** | 1.08 | 0.88 | 1.33 | 0.66 | 0.42 | 1.04 | 1.58* | 1.05 | 2.37 |

Notes: Time period was from 1 January 2009 to 31 May 2016. Results are shown from mixed-effect linear regression models for continuous outcomes and mixed-effect logistic regression models for binary outcomes. Independent variables included in the model are the following: practices’ early programme coverage of the NHS Health Check programme, year, and baseline age, gender, ethnicity, smoking status, BMI, antihypertensive medication, general practice IMD, and region.

DRS = diabetes risk score, NDH = non-diabetic hyperglycaemia, OR = Odds Ratio.

**p<0.01, *** p<0.001.

§ Tertiles of general practices coverage of the NHS Health Check Programme were defined based on programme coverage during the first three years after its implementation (2009-11).

**Table S5.** Differences in cardiovascular risk factors between 2009 and 2016 by general practices’ coverage of the NHS Health Check programme and individuals’ baseline diabetes risk score. Results obtained without adopting propensity score regression adjustment.

|  |  | **TOTAL SAMPLE** | | | **DRS ≥ 10** | | | |  |
| --- | --- | --- | --- | --- | --- | --- | --- | --- | --- |
| **SYSTOLIC BLOOD PRESSURE** | | **Coeff.** | **95% CI** | | | **Coeff.** | **95% CI** | | |
| **PROGRAMME COVERAGE^§^** | |  |  |  | |  |  | | |
| **Low** | | ref | | | | ref | | | |
| **Medium** | | -0.15** | -0.25 | -0.06 | | -0.38* | -0.75 | -0.01 | |
| **High** | | -0.39*** | -0.50 | -0.28 | | -0.70*** | -1.21 | -0.40 | |
| **DIASTOLIC BLOOD PRESSURE** | | **Coeff.** | **95% CI** | | | **Coeff.** | **95% CI** | | |
| **PROGRAMME COVERAGE^§^** | |  |  |  | |  |  | | |
| **Low** | | ref | | | | ref | | | |
| **Medium** | | -0.08* | -0.14 | -0.02 | | -0.20 | -0.42 | 0.03 | |
| **High** | | -0.09** | -0.16 | -0.03 | | -0.29* | -0.54 | -0.04 | |
| **BODY MASS INDEX** | | **Coeff.** | **95% CI** | | | **Coeff.** | **95% CI** | | |
| **PROGRAMME COVERAGE^§^** | |  |  |  | |  |  | | |
| **Low** | | ref | | | | ref | | | |
| **Medium** | | 0.08*** | 0.04 | 0.12 | | -0.08 | -0.22 | 0.05 | |
| **High** | | 0.25*** | 0.20 | 0.29 | | 0.10 | -0.06 | 0.25 | |
| **SMOKING PREVALENCE** | | **OR** | **95% CI** | | | **OR** | **95% CI** | | |
| **PROGRAMME COVERAGE^§^** | |  |  |  | |  |  | | |
| **Low** | | ref | | | | ref | | | |
| **Medium** | | 0.96* | 0.92 | 1.00 | | 0.95 | 0.82 | 1.10 | |
| **High** | | 1.08*** | 1.04 | 1.13 | | 0.97 | 0.80 | 1.16 | |
| **TOTAL CHOLESTEROL^¥^** | |  |  | | | **Coeff.** | **95% CI** | | |
| **PROGRAMME COVERAGE^§^** | |  |  |  | |  |  | | |
| **Low** | |  | | | | ref | | | |
| **Medium** | |  |  |  | | -0.01 | -0.05 | 0.02 | |
| **High** | |  |  |  | | -0.06** | -0.10 | -0.02 | |
| **STATINS^¥^** | |  |  | | | **OR** | **95% CI** | | |
| **PROGRAMME COVERAGE^§^** | |  |  |  | |  |  |  | |
| **Low** | |  |  |  | | ref | | | |
| **Medium** | |  |  |  | | 0.93 | 0.85 | 1.01 | |
| **High** | |  |  |  | | 1.05 | 0.96 | 1.15 | |
| **QRISK2** | | **Coeff.** | **95% CI** | | | **Coeff.** | **95% CI** | | |
| **PROGRAMME COVERAGE^§^** | |  |  |  | |  |  | | |
| **Low** | | ref | | | | ref | | | |
| **Medium** | | -0.09*** | -0.12 | -0.06 | | -0.12* | -0.23 | -0.01 | |
| **High** | | -0.14*** | -0.17 | -0.10 | | -0.26*** | -0.38 | -0.15 | |

Notes: Time period was from 1 January 2009 to 31 May 2016. Results are shown from mixed-effect linear regression models for continuous outcomes and mixed-effect logistic regression models for binary outcomes. Independent variables included in the model are the following: practices’ early programme coverage of the NHS Health Check programme, year, and baseline age, gender, ethnicity, smoking status, BMI, antihypertensive medication, general practice IMD, and region. Change in total cholesterol has been restricted to only those with a DRS ≥ 10 at baseline. DRS = diabetes risk score, NDH = non-diabetic hyperglycaemia, T2D = type 2 diabetes, OR = Odds Ratio.

*p<0.05, **p<0.01, *** p<0.001.

§ Tertiles of general practices coverage of the NHS Health Check Programme were defined based on programme coverage during the first three years after its implementation (2009-11).

¥ Differences in the mean total cholesterol and statins prescription have only been assessed for individuals at high risk of T2D at baseline because national guidelines do not recommend cholesterol monitoring for the whole population.

**Table S6.** Differences in cardiovascular disease risk factors in individuals with incident non-diabetic hyperglycaemia and type 2 diabetes after Jan 2009. Results obtained without adopting propensity score regression adjustment.

|  | **INCIDENT NON-DIABETIC HYPERGLYCAEMIA** | | | **INCIDENT TYPE 2 DIABETES** | | |
| --- | --- | --- | --- | --- | --- | --- |
| **SYSTOLIC BLOOD PRESSURE** | **Coeff.** | **95% CI** | | **Coeff.** | **95% CI** | |
| **PROGRAMME COVERAGE^§^** |  |  |  |  |  |  |
| **Low** | ref | | | ref | | |
| **Medium** | 0.26 | -0.42 | 0.95 | -0.51* | -1.01 | -0.12 |
| **High** | 0.26 | -0.48 | 1.01 | -0.90*** | -1.47 | -0.33 |
| **DIASTOLIC BLOOD PRESSURE** | **Coeff.** | **95% CI** | | **Coeff.** | **95% CI** | |
| **PROGRAMME COVERAGE^§^** |  |  |  |  |  |  |
| **Low** | ref | | | ref | | |
| **Medium** | 0.32 | -0.09 | 0.73 | -0.32* | -0.62 | -0.02 |
| **High** | 0.21 | -0.24 | 0.66 | -0.56** | -0.90 | -0.22 |
| **BODY MASS INDEX** | **Coeff.** | **95% CI** | | **Coeff.** | **95% CI** | |
| **PROGRAMME COVERAGE^§^** |  |  |  |  |  |  |
| **Low** | ref | | | ref | | |
| **Medium** | 0.06 | -0.20 | 0.33 | -0.10 | -0.31 | 0.12 |
| **High** | 0.19 | -0.09 | 0.47 | -0.01 | -0.23 | 0.25 |
| **SMOKING PREVALENCE** | **OR** | **95% CI** | | **OR** | **95% CI** | |
| **PROGRAMME COVERAGE^§^** |  |  |  |  |  |  |
| **Low** | ref | | | ref | | |
| **Medium** | 1.17 | 0.85 | 1.61 | 1.46** | 1.17 | 1.83 |
| **High** | 1.36 | 0.96 | 1.92 | 1.43** | 1.12 | 1.84 |
| **TOTAL CHOLESTEROL** | **Coeff.** | **95% CI** | | **Coeff.** | **95% CI** | |
| **PROGRAMME COVERAGE^§^** |  |  |  |  |  |  |
| **Low** | ref | | | ref | | |
| **Medium** | -0.03 | -0.09 | 0.02 | -0.02 | -0.07 | 0.03 |
| **High** | -0.10 | -0.16 | -0.04 | -0.04 | -0.10 | 0.01 |
| **STATINS** | **OR** | **95% CI** | | **OR** | **95% CI** | |
| **PROGRAMME COVERAGE^§^** |  |  |  |  |  |  |
| **Low** |  | ref |  |  | ref |  |
| **Medium** | 0.84 | 0.64 | 1.09 | 0.85 | 0.71 | 1.02 |
| **High** | 1.01 | 0.76 | 1.36 | 0.96 | 0.79 | 1.20 |
| **QRISK2** | **Coeff.** | **95% CI** | **Coeff.** | **95% CI** | **Coeff.** | **95% CI** |
| **PROGRAMME COVERAGE^§^** |  |  |  |  |  |  |
| **Low** |  | ref |  |  |  | ref |
| **Medium** | -0.27 | -0.71 | 0.17 | -0.33 | -0.77 | 0.10 |
| **High** | -1.14*** | -1.62 | -0.65 | -0.84** | -1.33 | -0.35 |

Notes: For the analyses were considered only individuals with incident non-diabetic hyperglycaemia and type 2 diabetes after Jan 2009. Individuals included in the analyses were followed-up from the year of diagnosis until the end of the study (May 2016). Results are shown from mixed-effect linear regression models for continuous outcomes and mixed-effect logistic regression models for binary outcomes. Independent variables included in the model are the following: practices’ early programme coverage of the NHS Health Check programme, year, and baseline age, gender, ethnicity, smoking status, BMI, antihypertensive medication, general practice IMD, and region. DRS = diabetes risk score, NDH = non-diabetic hyperglycaemia, T2D = type 2 diabetes, OR = Odds Ratio.

*p<0.05, **p<0.01, *** p<0.001.

§ Tertiles of general practices coverage of the NHS Health Check Programme were defined based on programme coverage during the first three years after its implementation (2009-11).

**SUPPLEMENTARTY INFORMATION FOR METHODS SECTION**

**Table S7.** List of Read codes used to identify individuals with non-diabetic hyperglycaemia

| **MEDCODE** | **READCODE** | **READTERM** |
| --- | --- | --- |
| 11050 | 44Uz.11 | Blood hyperglycaemia NOS |
| 19781 | 44V2.00 | Glucose tol. test impaired |
| 22959 | 66AJ000 | Chronic hyperglycaemia |
| 102389 | 8HlS.00 | Referral for management of impaired glucose tolerance |
| 106220 | 9m9..00 | Impaired glucose tolerance monitoring administration |
| 106316 | 9m90.00 | Impaired glucose tolerance monitoring invitation |
| 106273 | 9m90000 | Impaired glucose tolerance monitoring invitation 1st letter |
| 106275 | 9m90100 | Impaired glucose tolerance monitoring invitation 2nd letter |
| 106323 | 9m90200 | Impaired glucose tolerance monitoring invitation 3rd letter |
| 102668 | 9NS0400 | Referral for impaired glucose tolerance management offered |
| 10921 | C11y200 | Impaired glucose tolerance |
| 10983 | C11y300 | Impaired fasting glycaemia |
| 105434 | C11y400 | Impaired glucose regulation |
| 106604 | C11y500 | Pre-diabetes |
| 3505 | C313500 | Glucose intolerance |
| 11818 | R102.00 | [D]Glucose tolerance test abnormal |
| 11149 | R102.11 | [D]Prediabetes |
| 3295 | R102.12 | [D]Impaired glucose tolerance test |
| 1789 | R105712 | [D]Hyperglycaemia |
| 10791 | R10D000 | [D]Impaired fasting glycaemia |
| 31161 | R10D011 | [D]Impaired fasting glucose |
| 10042 | R10E.00 | [D]Impaired glucose tolerance |
| 9310 | Ryu8A00 | [X]Hyperglycaemia, unspecified |

**Figure S1.** Study tree showing final sample included in the current study.

**387,460** individuals aged 40-74 years between 2009 and 2014, living in England (**455** English general practices)

**38,473** individuals with a diagnosis of CVD or T2D before 2009

**348,987** individuals aged 40-74 years between 2009 and 2014, living in England and without a diagnosis of CVD or T2D before 2009 (**455** English general practices)

**Table S8.** Percentage of missing data for sub-group analyses on fasting plasma glucose and total cholesterol

| **FASTING PLASMA GLUCOSE** | **DRS ≥ 10** | **INCIDENT NON-DIABETIC HYPERGLYCAEMIA** | **INCIDENT TYPE 2 DIABETES** |
| --- | --- | --- | --- |
| **PROGRAMME COVERAGE** |  |  |  |
| **Low** | **16.6%** | **2.9%** | **3.7%** |
| **Medium** | **12.6%** | **2.0%** | **2.7%** |
| **High** | **10.9%** | **2.5%** | **3.4%** |
| **TOTAL** | **13.6%** | **2.4%** | **3.3%** |
| **HbA1c** |  |  |  |
| **PROGRAMME COVERAGE** |  |  |  |
| **Low** | **71.4%** | **23.8%** | **9.1%** |
| **Medium** | **66.0%** | **18.6%** | **6.5%** |
| **High** | **64.8%** | **17.6%** | **7.9%** |
| **TOTAL** | **67.6%** | **19.8%** | **7.8%** |
| **TOTAL CHOLESTEROL** |  |  |  |
| **PROGRAMME COVERAGE** |  |  |  |
| **Low** | **32.3%** | **2.6%** | **0.7%** |
| **Medium** | **27.3%** | **1.2%** | **0.4%** |
| **High** | **25.1%** | **1.3%** | **0.4%** |
| **TOTAL** | **28.5%** | **1.6%** | **0.5%** |
